# Gender Mediation in Adolescents’ Back Pain and Physical Fitness: A Cross-Sectional Study

**DOI:** 10.3390/healthcare10040696

**Published:** 2022-04-08

**Authors:** Noelia González-Gálvez, María Carrasco-Poyatos, Raquel Vaquero-Cristóbal, Pablo J. Marcos-Pardo

**Affiliations:** 1 Sport Injury Prevention Research Group, Faculty of Sports, Universidad Católica de Murcia (UCAM), 30107 Murcia, Spain; ngonzalez@ucam.edu; 2 Department of Education, Health and Public Administration Research Center, University of Almeria, 04120 Almeria, Spain; 3 Department of Education, Faculty of Education Sciences, University of Almeria, 04120 Almeria, Spain; pjmarcos@ual.es; 4 SPORT Research Group (CTS-1024), CERNEP Research Center, University of Almeria, 04120 Almeria, Spain

**Keywords:** adolescent, back pain, low back pain, exercise, physical fitness, paraspinal muscles

## Abstract

There is a lack of studies that analyze the interaction between risk variables as predictors of back pain (BP) in adolescents. The objectives of this study were to examine the relationship between BP and several risk variables, and to analyze the effect of the mediation of gender in this association. This cross-sectional study included *n* = 617 adolescents (mean age: 14.10 ± 1.18 years old) who completed the BP Adolescent Survey and who performed the bench trunk curl (BTC) and Sorensen (SOR) tests. Males showed a significantly lower prevalence (OR: 0.67) and frequency (contingency coefficient: 1.73) of BP than females, less participation in leisure-time sedentary behaviors (LRSBs) per day of more than 2 h (Cramer’s V: 0.110), a higher sufficient Physical Activity (PA) (Cramer’s V: 0.323) and a higher score in the BTC test (Contingency coefficient: 0.346). A high BTC score indicated significantly lower risk of BP than mid (OR: 1.74; *p* = 0.025) or low (OR: 1.62; *p* = 0.022) BTC. The mediation analysis showed a significant indirect effect with a significant value for the Sobel test (z = 7.45 ± 0.111). When the BTC test value was included in the equations, the connection between gender and BP was no longer significant. There was a difference in the prevalence between gender in BP and LRSB and PA. BP was associated with the SOR test. The association between BP and gender was mediated by SOR results.

## 1. Introduction

Low back pain (LBP) is the leading cause of disability globally [1], and the resulting economic burden generated is considered a worldwide problem among the population [2]. Furthermore, evidence suggests that the importance of cervical pain (CP) and thoracic spine pain (TSP) should also be analyzed in adolescence because the incidence and prevalence of pain in different areas of the spine are high in adolescents [3]. Therefore, spinal pain (SP) as a global concept that encompasses CP, TSP, and LBP could be key during adolescence [3]. Previous studies have shown different figures of SP prevalence, ranging from 10.7% to 78.5% [4,5], with this prevalence growing worldwide [3].

Several studies have shown that a large proportion of the SP in adolescents is idiopathic, without an anatomic pathology, suggesting that the pain patterns should be established before adulthood for reducing its incidence [3]. Therefore, several research studies have been performed to assess the causes and risk factors of SP in adolescents [3].

Some studies associate a higher prevalence of SP to females than males [4,6,7,8], although other investigations do not show this association [3,9]. On the other hand, males usually show a higher frequency or duration of physical activity (PA) [6,10], lower number of hours in leisure-time sedentary behaviors (LRSBs) [4], and different trunk muscle endurance [1,8,11,12] than females. At the same time, the influence of PA on physical fitness has been previously shown [13].

Considering that some research studies have described a correlation between SP and PA [9], LRSB [4,5,6,9,14] or trunk muscle endurance [1,7], these variables could be confounding the association between SP and gender. Therefore, it could be that the differences in the incidence of back pain between genders are not entirely due to their morphological characteristics, but to the differences between genders in the way they spend their free time and the influence of the practice of physical activity on some of the factors related to back pain, such as the resistance of the trunk musculature. If this is the case, interventions could be carried out in adolescents to improve these parameters and reduce the incidence of back pain, independently of gender. Nevertheless, no research study has assessed the influence of physical conditioning or sedentary behaviors on SP or monitored the interaction with gender, and only a few studies have monitored PA by examining the interaction between different variables [9].

Thus, a need was detected for a research study to analyze the influence of physical fitness on SP, monitoring gender and the prevalence of PA and LRSB. Therefore, the purposes of the present study are (a) to determine the prevalence of SP in a sample of adolescents; (b) to examine the relationship between SP and gender, LRSB, PA and trunk flexor and extensor endurance by monitoring the interaction among the variables; and (c) to examine the effect of the mediation of gender in the association between back pain and the endurance of the trunk musculature. The hypotheses of the present study were (a) there is a high prevalence of SP among adolescents, especially low back pain; (b) females have more SP, spend more time in sedentary behaviors, have lower levels of PA, and have lower trunk muscle endurance; and (c) an important part of the difference in the incidence of SP between males and females is due to the behavioral characteristics regarding the sedentary behavior, practice of PA, and physical fitness of adolescent females.

## 2. Materials and Methods

### 2.1. Design

This is a cross-sectional study that took place in high schools located in the Region of Murcia (Spain). The institutional research ethics board approved the study protocol, and adolescents and their parents or legal guardians signed an informed consent form approved by the scientific and ethical committee, conducted in accordance with the Declaration of Helsinki (Universidad Católica San Antonio, number of the ethics protocol: EC101701). The trial design was registered with ClinicalTrials.gov (identifier: NCT03831867). The cross-sectional study design followed the Strobe Statement.

### 2.2. Participants

The participants were volunteer adolescents aged 12 to 17 years old (mean age: 14.10 ± 1.18 years old). A total of three high schools were invited to participate in the study from September 2016 to October 2016. The inclusion criteria to participate in the study were (a) being enrolled in the 1st to 4th years of Secondary Education, (b) being present on assessment day, and (c) not having any musculoskeletal, neurological, cardiovascular, metabolic, or rheumatic alterations different from SP that would prevent the students from living a normal life. The exclusion criteria were (1) not being authorized to participate in the measurements and (2) not having completed all the assessments included in this study.

The calculations to establish the sample size were performed using Rstudio 3.15.0 software. The significance level was set at α = 0.05. According to the standard deviation established for the Sorensen test (SOR) in previous studies [15] and an estimated error of 6s, a valid sample size of 439 was needed for a confidence interval of 95%. A total of 617 students completing the trial would provide a power of 95% if between and within a variance of 5.06 s.

In addition, the students’ mean body mass index value in this sample was similar to those found in the results from the HBSC-2014 study in Spain [16]. Therefore, the subjects of this study were defined as being representative of adolescents in Spain as a whole, as shown in Kyan, Takakura, and Miyagi [17].

### 2.3. Procedures

Assessments tests were performed by the same examiners in a single session between the hours of 10:00 and 14:00 from September 2016 to October 2016. They were performed without a previous warm-up, with bare feet, and at random. There was a 5 min rest between measurements. Before the examination, to establish the reliability of the examiner, a double-blind study was performed with 30 subjects, obtaining an intraclass correlation coefficient higher than 95%.

The Back Pain Adolescent Survey, used to discover the prevalence of SP, PA, and sedentary time, was designed and validated by Martínez-Crespo et al. [5] with a kappa coefficient >0.75. The survey asked adolescents about SP, CP, TBP, and LBP. For SP, a visual representation of a human silhouette was used for patients to indicate the site of their pain. The use of this device is advantageous for younger participants, who sometimes have difficulties in describing their pain [18]. The SP during the past year was determined as follows: “having SP during the past year that hampered or limited activities at school or in their leisure time for more than three months”. Being insufficiently active was considered as practicing less than seven hours per week of moderate or vigorous PA [13]. Sedentary time in their leisure time was defined as activities such as sitting, lying down, watching TV, reading, doing homework, and so on; except for the time spent sleeping at night. Sedentary time was divided into less or equal to two hours per day and more than two hours per day, following the recommendation of the Physical Activity Guidelines Advisory Committee [19]. The adolescents were supervised while completing the questionnaires, and they were reviewed immediately after completion.

Body mass was measured using a SECA 762 scale (SECA, Germany) and height using a GPM anthropometer (Siber-Hegner, Switzerland). After that, body mass index (BMI) was calculated with the Quetelet Index formula (BMI = weight (kg)/height (m^2^) [20].

The bench trunk curl (BTC) test was used to evaluate trunk flexor endurance. This test is safe, protects the back, and isolates the abdominal musculature. Its reliability and validity have been demonstrated elsewhere (women: r = 0.94; men: r = 0.88) [21], and it has been used in previous research studies with adolescents [22]. The subject was placed in the supine position with legs on top of a chair that was 0.46 m in height, in such a way that the knees and the hips stayed at a 90° angle. The arms were crossed over the chest. The subject curled the trunk so that the forearms touched the front of the thigh and finished the movement by touching the ground with the scapula. Subjects had to repeat this movement for 120 s. The total number of repetitions was recorded [21].

Trunk extensor endurance was measured using the SOR test. Its reliability has already been established elsewhere for adolescents (ICC = 0.94–0.999) [23,24], and it has been previously used in research studies with adolescents [15,16,17]. This test was chosen to ensure the isolation of the trunk extensor musculature, as opposed to other dynamic tests [22]. The subject was placed in the prone position on the examination table, with the upper border of the iliac crests aligned with the edge of the table. The lower part of the body was held by an auxiliary person. With the arms crossed behind the back, the subject was asked to keep the upper part of the body horizontal until they could no longer support the position. The total number of seconds was noted [22,25].

### 2.4. Statistical Analysis

The normality of the data was evaluated using the Kolmogorov–Smirnov test, and Mauchly’s W-test was used to analyze the normality and the sphericity of the data. A descriptive analysis was performed for the quantitative variables (means and standard deviations) and qualitative variables (frequency). The results of the BTC and SOR tests were categorized as low (25%), mid (50%), or high (25%) for the analysis [8]. Low was considered as the worst value for each test, and high the best value. A X^2^ test (categorical variables) was used to analyze the differences between groups. A Cramer’s V post hoc comparison was applied for 2 × 2 tables, and for 2 × n tables, a contingency coefficient was applied, showing the value of the statistic and the *p* value. The maximum expected value was 0.707, with an r < 0.3 showing a low association, a moderate association defined as an r value between 0.3 and 0.5, and a high association defined as r > 0.5. Logistic regression analyses were used to estimate the associations between the dependent variables and each independent variable. A multiple logistic regression analysis was performed to examine the association of BTC level and SOR level with the frequency of SP. The results were reported as raw and adjusted odds ratios (ORs) with 95% confidence intervals (CIs). Potential confounders were selected based on a previous study. In addition, adjustments for gender, PA, and LRSB were performed. The 95% CI of the odds ratios was set to express the magnitude of the associations. The mediation analysis was performed by Process macro for SPSS (SPSS Inc., Chicago, IL, USA). A classical Baron and Kenny step regression method was used [26]. To test if the mediation effect had statistical significance, the Sobel test was used [27]. If the association between dependent and independent variables disappeared after the mediation variable was included, the mediation variable was considered as a complete mediator. The statistical analysis was performed using IBM SPSS Statistics (version 25.0). An error of *p* ≤ 0.05 was set.

## 3. Results

The baseline characteristics of the adolescents are shown in Table 1. A total of 617 adolescents (male = 354; female = 263) with the mean (SD) age of 14.10 (±1.2) years were enrolled in the study. The SP, CP, TBP, and LBP during the past year were 30.4%, 3.73%, 12.16%, and 20.26%, respectively.

The male subjects (MS) showed significantly lower prevalence of SP than the female subjects (FS) (MS: 26.84; FS: 35.36; OR: 0.67; *p* = 0.023), lower prevalence of CP (MS: 2.26; FS: 5.7; OR: 0.38; *p* = 0.031) (Table 1 and Table 2), lower SP frequency (contingency coefficient: 1.73; *p* = 0.01) (Table 1), less participation in sedentary time per day over 2 h (MS: 24.86%; FS: 34.98%; Cramer’s V: 0.110; *p* = 0.006) (Table 1 and Table 2), higher sufficient PA (MS: 35.88; FS: 7.98; Cramer’s V: 0.323; *p* < 0.001) and higher score in the BTC test (MS: 61.04; FS: 45.38; contingency coefficient: 0.346; *p* < 0.001) (Table 1).

The highest scores for the BTC test were found to be significant (*p* < 0.001) for MS (61.04 ± 29.1) compared to the FS (45.38 ± 19.9); and significant (*p* < 0.001) for sufficiently active adolescents (67.90 ± 33.1) compared to insufficiently active ones (50.09 ± 22.8) (Table 3). Having a high BTC significantly indicated lower risk of SP than mid (OR:1.74; *p* = 0.025) or low BTC (OR:1.62; *p* = 0.022) (Table 4). A lower BTC level was significantly associated with high SP frequency (often/usually), as compared with a higher BTC level (OR: 2.637 (1.031; 6.744); *p* = 0.043). Whether the model was adjusted for sufficient (≥7 h/week) or insufficient (<7 h/week) PA, this association slightly changed (OR: 3.129 (1.180; 8.296) *p* = 0.022) (Table 5).

A high SOR was associated with lower prevalence of SP than mid SOR (SOR: 1.77; *p* = 0.023) or low SOR (SOR: 1.48; *p* = 0.059), significantly and with a tendency towards significance, respectively (Table 4). The lower SOR category was significantly associated with SP frequency (rarely/sometime) (OR: 1.844 (1.060; 3.209); *p* = 0.03) as compared with a higher SOR category (Table 5).

**Figure 1:**
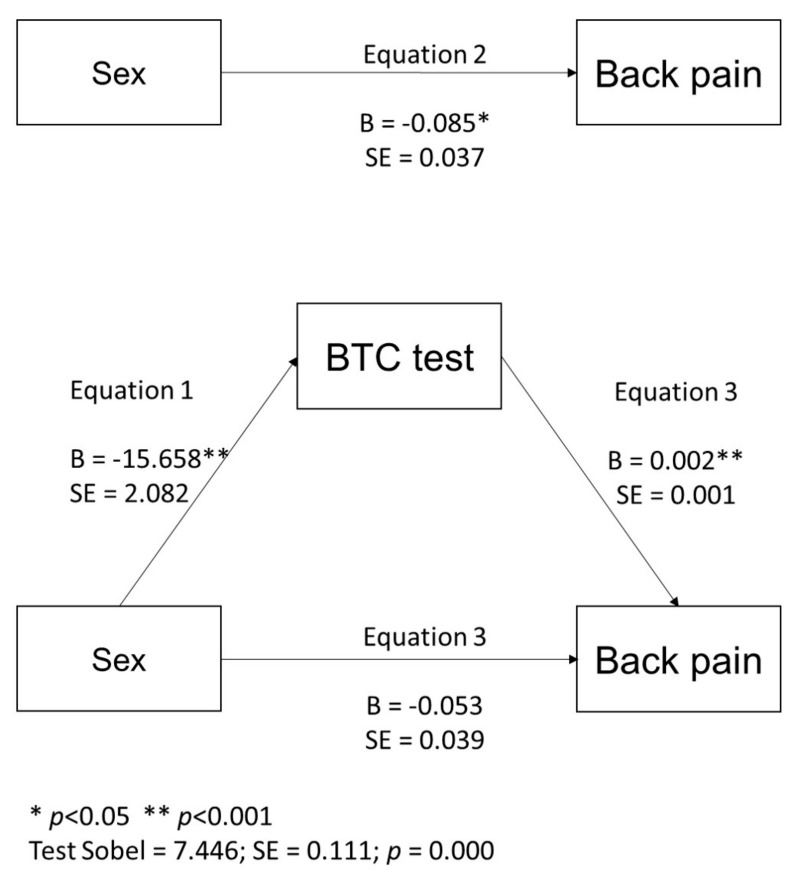
Mediation of gender and SP by BTC test.

The mediation analysis showed a significant indirect effect with a significant value for the Sobel test (z = 7.45 ± 0.111; *p* < 0.001). When the BTC test value was included in the equations, the association between gender and back pain was no longer significant (Figure 1).

## 4. Discussion

In the present study, a high prevalence of SP was observed in adolescents in the past year. The results showed, as in other research studies, that the area of the body with a high prevalence was the lumbar region [6,18], with a low prevalence found in the cervical region [5,18]. Previous research shows a different prevalence of SP results among adolescents, ranging from 10.7% to 78.5% [4,5,18]. These differences in the prevalence of pain are the result of the different periods of time studied (from one week to lifetime), the areas included [4], and/or characteristics of the sample utilized for each research study. For example, the highest prevalence was shown by Martínez-Crespo et al. [5], with their sample showing a larger percentage of more than two hours of sedentary activity (44.9%), an aspect that could be related.

Females showed a significantly higher prevalence of SP than males, as other studies have shown for general SP [3,6,18], CP [3], TBP [4], and LBP [6,7]. Males showed a higher prevalence of PA than females, as in other studies [8,9,11]. However, the results of the present investigation did not show the presence of a greater risk of suffering from any SP for the subjects considered inactive. Some research studies have not described a connection between PA and SP [7,14], and others have indicated a connection between these variables [5,9]. However, when the association between the BTC level and SP frequency was adjusted for sufficient PA or insufficient PA, the association slightly changed, suggesting that the frequency of PA has an influence on SP frequency. This result, in addition to the other research studies, provides a novel finding, which indicates that despite PA being an influencing variable, it is not the most important variable for SP; PA could be more relevant to SP frequency than SP prevalence. On the other hand, the type, intensity, frequency, and duration of PA must be considered in terms of which one is the most beneficial to the health of SP. It has been described that a moderate level and endurance-based PA is associated with reduced SP, and as Guddal et al. [6] showed, one must consider the type, level, and intensity of PA, not only the prevalence of PA, when evaluating SP.

Regarding fitness condition, the present study showed that females had a lower score of trunk flexor endurance than males. Likewise, the adolescents with poor trunk flexor endurance showed a higher risk of SP than the rest. In addition, the difference in SP prevalence between genders ceased to be significant after adjusting for trunk flexor endurance, and the mediation analysis indicated that this association was entirely mediated by trunk endurance. In addition, a new finding from this study indicates that trunk flexor endurance levels are connected with the frequency of SP. Other studies have also reported on the relevance of frequency and level of pain to other variables [28]. This supports the importance of the trunk flexor endurance on SP. In this sense, several studies have associated SP with low trunk flexor endurance [8,11,12], and it has been shown that trunk endurance is different between genders and is moderately determined by genes [8,12]. We could indicate that trunk flexor endurance is the factor that influences a higher SP, and the different physical conditions according to gender explain the different prevalence of pain. In this way, coinciding with the conclusions by Calvo-Muñoz et al. [3], it could be concluded that there is no significant correlation between SP and gender, and this connection is due to the interaction with trunk flexor endurance.

Subjects with a high trunk extensor endurance showed a lower risk of SP than the rest. In addition, the trunk extensor endurance level is connected with the frequency of SP. Many research studies have analyzed the relationship between SP and trunk extensor endurance, showing an inverse correlation [1,11,12], while other studies have associated SP with low trunk extensor endurance [12] in adolescents. However, other studies [8] have not correlated trunk extensor endurance with SP, although these authors inquired about SP in the past month, instead of the past year as is used for most studies. In this sense, Bernard et al. [11] indicated that there was a greater risk in chronic/diagnosed subjects than those with undiagnosed recurrent SP. Therefore, the non-presence of this relationship in Perry et al. [8] may be due to the importance of the frequency of SP, taking into account at least three months of SP, as in the present research. This is in agreement with the present findings, which connect SP frequency with trunk extensor endurance level.

Conversely, these study results should be considered in light of several limitations. The reduced sample of 617 adolescents, although similar to [5] or more numerous than in other research studies [11], limits the ability to extrapolate the results. Because of this, the results are not generalizable to other adolescent populations. Furthermore, as this is the first study to examine this issue, future studies need to confirm the findings of the present research. Another limitation is that, although the questionnaire was a valid instrument for adolescents, it has to been taken into account that the variety of frequency and insensitivity to SP could influence the results of the study. In addition, another limitation is that there are other parameters that have been shown to have a significant influence on the incidence of SP. These include the type of sport practiced [29], differences in sagittal spine disposition and pelvic tilt [9], hamstring extensibility [9], anthropometric variables [9,18], or specific sedentary leisure activities. For the latter, the daily time spent on electronic devices such as computers, cell phone, and tablets [30,31] has shown different values depending on gender [9,29,30,31]. Lastly, other factors such as a reported family history of LBP [32], age [9], and pubertal development [18] could also be modulating factors in the difference between genders in the incidence of SP. As a consequence, future studies should include the modulating variables that have been included in the present research, in addition to the variables detected by previous research, in order to analyze what are, from a multifactorial point of view, the factors that modulate the difference in the incidence of SP between genders.

## 5. Conclusions

Almost one-third of the sample had SP, and the region with a high prevalence was the lumbar region. Trunk flexor endurance (BTC test) and trunk extensor endurance (SOR test) were considered as a risk factor for SP. Males showed a significantly lower prevalence and frequency of BP than females, less participation in LRSB per day of more than 2 h, a higher sufficient PA, and a higher score in the BTC test. When the BTC test value was included in the equations, the connection between gender and BP was no longer significant. The practical implications of these results are that regardless of gender, increasing the strength of trunk flexor and extensor muscles could decrease the incidence of SP in adolescents.

## Figures and Tables

**Table 1:** Basic characteristics of the adolescents.

	Total (*n* = 617)	Male (*n* = 354)	Female (*n* = 263)	*p* Value
	Mean ± SD	Mean ± SD	Mean ± SD	
Age	14.1 ± 1.2	14.06 ± 1.2	14.14 ± 1.1	0.391
BMI	22.43 ± 4.5	22.38 ± 4.0	22.50 ± 5.2	0.749
	**% (*n*)**	**% (*n*)**	**% (*n*)**	***p* value**
Gender				
Male	57.37 (354)			
Female	42.63 (263)			
Pain				
SP	30.47 (188)	26.84 (95)	35.36 (93)	0.023
CP	3.73 (23)	2.26 (8)	5.70 (15)	0.031
TBP	12.16 (75)	11.02 (39)	13.69 (36)	0.315
LBP	20.26 (125)	17.80 (63)	23.57 (62)	0.077
SP frequency				
Never	69.53 (429)	73.16 (259)	64.64 (170)	0.001
Rarely/Sometime	23.34 (144)	21.47 (76)	25.86 (68)	
Often/Usually	7.13 (44)	5.37 (19)	9.51 (25)	
PA				
Insufficient (<7 h/week)	76.01 (469)	64.12 (227)	92.02 (242)	0.000
Active (≥7 h/week)	23.99 (148)	35.88 (127)	7.98 (21)	
Leisure-time sedentary behaviours (Time: television, computer, or video games)				
≤2 h/day	70.83 (437)	75.14 (266)	65.02 (171)	0.006
>2 h/day	29.17 (180)	24.86 (88)	34.98 (92)	
BTC				
Low	24.15 (149)	13.28 (47)	38.78 (102)	0.000
Mid	51.05 (315)	51.98 (184)	49.81 (131)	
High	24.80 (153)	34.75 (123)	11.41 (30)	
SOR				
Low	24.84 (153)	22.03 (78)	28.63 (75)	0.100
Mid	51.14 (315)	54.52 (193)	46.56 (122)	
High	24.03 (148)	23.45 (83)	24.81 (65)	

Note: BMI = Body Mass Index; SP = spinal pain; CP = Cervical pain; TBP = Thoracic back pain; LBP = Low back pain; PA = physical activity; BTC = Bench trunk curl test; SOR = Sorensen test.

**Table 2:** Association between SP and gender, PA level, and leisure-time sedentary behaviors unadjusted, adjusted by gender, and adjusted by PA.

	Presence of Pain % (*n*) 30.47% (*n* = 188)	Absence of Pain % (*n*) 69.53% (*n* = 429)	Unadjusted		Adjusted by Gender		Adjusted by PA	
			OR	*p* Value	OR	*p* Value	OR	*p* Value
Gender								
Male	26.84 (95)	73.16 (259)	0.67	0.023	-	-	0.63	0.014
Female	35.36 (93)	64.64 (170)	1		-		1	
PA								
Insufficient (<7 h/week)	30.66 (65)	69.34 (147)	0.96	0.853	0.81	0.320	-	-
Active (≥7 h/week)	30.37 (123)	69.63 (282)	1		1		-	
Leisure-time sedentary behaviours (Time: television, computer or video games)								
≤2 h/day	29.98 (131)	70.02 (306)	0.92	0.679	0.97	0.869	0.92	0.662
>2 h/day	31.67 (57)	68.33 (123)	1		1		1	

**Table 3:** Descriptive data of the tests (BTC and SOR) and their association with gender and PA.

Gender					
	**Male (*n* = 354)** **Mean ± SD**	**Female (*n* = 263)** **Mean ± SD**	**Dif. M-F**	**CI 95% (MM-MF)**	***p* Value**
BTC	61.04 ± 29.1	45.38 ± 19.9	15.66	11.57; 19.75	˂0.001
SOR	130.25 ± 71.6	127.54 ± 73.9	2.71	−8.91; 14.33	0.647
PA					
	**Insuffiently (*n* = 469)** **Mean ± SD**	**Active (*n* = 148)** **Mean ± SD**	**Dif. Insuf-Active**	**CI 95% (MInsuf-MActive)**	***p* Value**
BTC	50.09 ± 22.8	67.90 ± 33.1	−17.81	−23.56; −2.07	˂0.001
SOR	121.32 ± 71.8	153.68 ± 69.6	−32.36	−45.56; −9.16	˂0.001

Note: PA = physical activity; BTC = Bench trunk curl test; SOR = Sorensen test.

**Table 4:** Association between SP and BTC and SOR test unadjusted, adjusted by gender, and adjusted by PA.

	Presence of Pain % (*n*) 30.47%; (*n* = 188)	Absence of Pain % (*n*) 69.53%; (*n* = 429)	Unadjusted		Adjusted by Gender		Adjusted by PA	
			OR	*p* Value	OR	*p* Value	OR	*p* Value
BTC								
Low	38.93 (58)	61.07 (91)	1.62	0.022	1.50	0.060	1.64	0.019
Mid	28.25 (89)	71.75 (226)	1.74	0.025	1.51	0.120	1.82	0.020
High	26.80 (41)	73.20 (112)	1		1		1	
SOR								
Low	37.91 (58)	62.09 (95)	1.48	0.059	1.43	0.090	1.52	0.047
Mid	29.21 (92)	70.79 (223)	1.77	0.023	1.74	0.028	1.85	0.018
High	25.68 (38)	74.32 (110)	1		1		1	

Note: PA = physical activity; BTC = Bench trunk curl test; SOR = Sorensen test.

**Table 5:** Association of BTC and SOR level with the frequency of SP.

Frequency of SP		Low	Mid	High
BTC Level				
Rarely/Sometimes	% (*n*)	28.86 (43)	21.27 (67)	22.22 (34)
	OR (95% CI)	1.557 (0.918; 2.639)	0.977 (0.610; 1.564)	1
	*p* value	0.101	0.921	
Often/Usually	% (*n*)	10.07 (15)	6.98 (22)	4.58 (7)
	OR (95% CI)	2.637 (1.031; 6.744)	1.558 (0.646; 3.756)	1
	*p* value	0.043	0.324	
SOR Level				
Rarely/Sometimes	% (*n*)	28.10 (43)	23.49 (74)	18.24 (27)
	OR (95% CI)	1.844 (1.060; 3.209)	1.352 (0.823; 2.221)	1
	*p* value	0.030	0.234	
Often/Usually	% (*n*)	9.80 (15)	5.71 (18)	7.43 (11)
	OR (95% CI)	1.579 (0.692; 3.603)	0.807 (0.369; 1.768)	1
	*p* value	0.278	0.592	

Note: SP = spinal pain; BTC = Bench trunk curl test; SOR = Sorensen test.

## Data Availability

MDPI Research Data Policies.
